# Association between obesity and the prevalence of dyslipidemia in middle-aged and older people: an observational study

**DOI:** 10.1038/s41598-024-62892-5

**Published:** 2024-05-25

**Authors:** Chuanlei Zheng, Yanhong Liu, Cong Xu, Shaobo Zeng, Qi Wang, Yixing Guo, Jian Li, Sisi Li, Minghua Dong, Xiaoting Luo, Qingfeng Wu

**Affiliations:** 1 School of Public Health and Health Management, Gannan Medical University, Ganzhou, 341000 China; 2 School of Basic Medicine, Gannan Medical University, Ganzhou, 341000 China; 3 Division of Academic Affairs, Gannan Medical University, Ganzhou, 341000 China

**Keywords:** Obesity, Body mass index, Waist circumference, Dyslipidemia, Restricted cubic spline, Dyslipidaemias, Obesity, Epidemiology

## Abstract

This study aimed to explore the link between various forms of obesity, including body mass index (BMI) and waist circumference (WC), and the risk of dyslipidemia among Chinese residents. We selected the study population through a multi-stage random sampling method from permanent residents aged 35 and older in Ganzhou. Obesity was categorized as non-obesity, general obesity, central obesity, or compound obesity according to established diagnostic criteria. We employed a logistic regression model to assess the relationship between different types of obesity and the risk of dyslipidemia. Additionally, we used the restricted cubic spline model to analyze the association between BMI, WC, and the risk of dyslipidemia. The study included 2030 residents aged 35 or older from Ganzhou, China. The prevalence of dyslipidemia was found to be 39.31%, with an age-standardized prevalence of 36.51%. The highest prevalence of dyslipidemia, 58.79%, was observed among those with compound obesity. After adjusting for confounding factors, we found that the risk of dyslipidemia in those with central and compound obesity was respectively 2.00 (95% CI 1.62–2.46) and 2.86 (95% CI 2.03–4.03) times higher than in the non-obese population. Moreover, the analysis using the restricted cubic spline model indicated a nearly linear association between BMI, WC, and the risk of dyslipidemia. The findings emphasize the significant prevalence of both dyslipidemia and obesity among adults aged 35 and above in Ganzhou, China. Notably, individuals with compound obesity are at a substantially increased risk of dyslipidemia. Therefore, it is crucial to prioritize the use of BMI and WC as screening and preventive measures for related health conditions.

## Introduction

With the rapid economic growth in China, residents' dietary patterns, behavioral habits, and lifestyles have seen significant transformations. The traditional plant-based diet, primarily composed of whole grains and vegetables, has shifted toward a more Westernized dietary pattern^1^. This change, combined with decreased physical activity and increased sedentary behavior^2^, has exacerbated obesity^3^. Central obesity is a major concern^4^. A comparative analysis of two national cross-sectional surveys conducted between 2007–2008 and 2015–2017 revealed a 5.3% increase in the prevalence of general obesity and a 9.5% increase in central obesity over the decade^5^. These trends are placing a significant burden on the national healthcare system. A domestic study conservatively estimated that by 2030, obesity-related medical expenses could account for up to 22% of total medical costs^6^. Obesity poses a substantial threat to human health and is recognized by the World Health Organization as one of the top ten chronic diseases. It also increases the risk of conditions such as dyslipidemia, hypertension, diabetes, coronary heart disease, and myocardial infarction^4^.

Dyslipidemia typically involves elevated levels of total cholesterol (TC), triglycerides (TG), and low-density lipoprotein cholesterol (LDL-C), alongside reduced levels of high-density lipoprotein cholesterol (HDL-C)^7^. Recently, there has been a noticeable shift in lipid levels within the Chinese population, marked by a gradual increase in the prevalence of dyslipidemia. Data from a national survey conducted in 2002 revealed that the average serum levels of TC, LDL-C, and TG in Chinese adults were 3.93 mmol/L, 2.12 mmol/L, and 1.12 mmol/L, respectively^8^. These lipid levels have since shown significant increases, rising to 4.8 mmol/L for TC, 2.9 mmol/L for LDL-C, and 1.7 mmol/L for TG^9^. The China Million Population Project, conducted from 2014 to 2019, focused on early screening and comprehensive intervention for high-risk individuals with cardiovascular disease. This project reported a dyslipidemia prevalence of 33.8% among adults aged ≥ 35 years^10^, which is notably higher than the 18.6% rate reported in 2002^11^. An international study projected an increase of approximately 9.2 million cardiovascular events due to dyslipidemia in the Chinese population between 2010 and 2030^12^.

Previous studies have confirmed that obesity is a significant risk factor for dyslipidemia^7^. Research indicates that insulin resistance may be the underlying mechanism linking obesity to dyslipidemia. Insulin plays a role in breaking down TG-rich lipoproteins. When insulin resistance occurs, the clearance of these lipoproteins from the bloodstream is impaired, leading to increased TG levels. Elevated TG levels can trigger cholesteryl ester transfer protein activity, resulting in the production of TG-rich high-density lipoprotein particles that are susceptible to hydrolysis by plasma lipases, ultimately reducing HDL-C levels^13^. The relationship between obesity and dyslipidemia is not only influenced by overall obesity but is also closely linked to central obesity^13–16^. In particular, the prevalence of dyslipidemia in the general obese population is 3.0 times higher than that in the normal population, while the prevalence of elevated hypertriglyceridemia and low HDL-C in the central obesity population is 2.5 times and 1.8 times higher than that in healthy figures, respectively^17^. Current research, both domestically and internationally, has primarily focused on the relationship between types of obesity defined by a single indicator and dyslipidemia. However, there is a lack of comprehensive studies exploring the association between compound obesity and dyslipidemia. This study aimed to investigate the correlation between various types of obesity, including body mass index (BMI) and waist circumference (WC), and the risk of dyslipidemia in permanent residents over 35 years old in southern Jiangxi. Additionally, this study sought to assess the impact of different types of obesity on blood lipid levels and to provide intervention strategies focusing on a balanced diet, physical activity, and health education. This research serves as a foundation for the development of evidence-based prevention and control policies for obesity and dyslipidemia among residents in southern Jiangxi.

## Materials and methods

### Study design

This study used a multistage sampling method to determine the required sample size. The formula used was $n=\frac{{Z}_{1-\frac{\alpha }{2}}^{2}p(1-p)}{{d}^{2}}\times Deff$, where *p* is the expected prevalence rate, *d* is the allowable error, *α* is the test level, and *Deff* is the design effect. For this study, *α* was set at 0.05, *p* was 33.8% based on a national investigation in China^10^, *d* was 0.1*p*, *Deff* was 2, and the expected response rate was set at ≥ 85%, then a sample size of 1734 individuals was deemed necessary. The data in this study were collected as the baseline of the Gannan Medical University cohort study conducted from 2022 to 2023. Multistage sampling method was used to collect participations in Ganzhou City. According to the varying levels of economic development, the 18 counties in Ganzhou are classified into high, medium, and low categories. From each category, 1–3 counties are chosen at random. Subsequently, 1–3 communities or streets are randomly selected from the chosen counties, with approximately 300 individuals sampled from each community or street. Questionnaires, physical examinations, and biochemical index tests were administered to all 2131 participants in the selected units. After applying inclusion and exclusion criteria, 2030 valid samples were included. The inclusion criteria were: (1) local residents aged 35 years or older; (2) absence of mental illness and ability to cooperate with the investigation; and (3) signed informed consent. The exclusion criteria were: (1) secondary obesity; (2) severe heart, brain, kidney, or other fundamental diseases; (3) pregnancy; (4) incomplete physical examination indicators; and (5) incomplete biochemical indicators. This study adhered to the principles of the Declaration of Helsinki and was approved by the Medical Ethics Committee of Gannan Medical University (Ethics Committee Number: No.2019129). All participants volunteered for the study and provided written informed consent.

### Information and biomarkers collection

This study primarily involved conducting questionnaire surveys, performing physical examinations, and collecting and analyzing biochemical indicators. Questionnaires were administered through face-to-face interviews using self-compiled questionnaires that covered general health conditions, smoking and drinking habits, physical activity, disease history, and other relevant factors. The physical examination focused on measurements of height, weight, and WC. Blood samples were collected after an overnight fast, between 7 a.m. and 10 a.m., and were transported to the physical examination center for laboratory analysis within 12 h. Biomarkers were analyzed using a Beckman Coulter AU5800 fully automatic biochemical analyzer. Techniques such as latex particle-enhanced turbidimetry, hexokinase method, GPO-PAP, CHOD-PAP, catalase scavenging, and surfactant scavenging were employed to detect glycosylated hemoglobin (HbA1c), fasting plasma glucose (FPG), TG, TC, HDL-C, and LDL-C. The kits used for these detections were supplied by Medical System company. Questionnaires and physical examinations were administered by investigators who had received consistent training. All samples were processed by skilled medical personnel in the laboratory department.

### *Indicator* definitions

BMI was calculated using the following formula: BMI = weight/(height)^2^. According to the obesity classification standards outlined by Zhang Siting et al^18^, non-obese individuals are characterized by a BMI < 28.0 kg/m^2^ and a WC < 90.0 cm for men and < 85.0 cm for women. General obesity is defined as a BMI ≥ 28.0 kg/m^2^ with a WC < 90.0 cm for men and < 85.0 cm for women. Central obesity is identified by a BMI < 28.0 kg/m^2^ and a WC ≥ 90.0 cm for men and ≥ 85.0 cm for women. Compound obesity is defined as both a BMI ≥ 28.0 kg/m^2^ and a WC ≥ 90.0 cm for men and ≥ 85.0 cm for women.

Dyslipidemia is defined as having TC ≥ 6.22 mmol/L, LDL-C ≥ 4.14 mmol/L, HDL-C ≤ 1.04 mmol/L, TG levels ≥ 2.26 mmol/L, or self-reported use of lipid-lowering medications, in accordance with the 2016 Chinese Adult Dyslipidemia Prevention Guideline^7^. Type 2 diabetes, as defined by the American Diabetes Association^19^, is characterized by FPG levels ≥ 7.0 mmol/L and/or glycosylated hemoglobin levels ≥ 6.5%, or current use of antidiabetic drugs. Smoking is defined as the habitual consumption of at least one cigarette daily for more than six months; quitting is defined as reducing smoking to less than one cigarette per week for more than three months. Passive smoking occurs when a non-smoker inhales smoke exhaled from a smoker for at least 15 min daily. Drinking is defined as the regular consumption of alcohol on more than 12 occasions within a year, while non-drinking refers to consuming alcohol fewer than 12 times annually.

### Quality control measures

Before conducting an epidemiological study, a preliminary investigation is necessary to identify and address potential issues. Comprehensive training for investigators is organized to clarify the objectives and procedures of the study and to standardize the completion of relevant forms. Residents are advised to consume light meals three days before the physical examination, avoid overeating and strenuous exercise, and maintain an empty stomach on the examination day. During questionnaire collection, any missing information was promptly addressed.

To ensure the accuracy of the test specimen, the entire testing process is strictly conducted in accordance with instrument and test kit requirements. Prior to testing, an estimation of reagent quantity is made to guarantee sufficient dosage for a single addition. Subsequently, laboratory quality control tests are performed using standard samples (quality control reagents sourced from the American Bio-rad Company), followed by formal blood sample testing once normal results are obtained. Trained technicians carry out the testing procedure in strict adherence to standard operating procedures, while continuously monitoring blood sample results to promptly identify and address any abnormalities.

### Statistical analysis

Statistical Product and Service Solutions version 14.0 (SPSS 14.0) was used for data cleaning and analysis. Categorical variables were presented as constituent ratios or rates and analyzed using the* χ*^2^ test. A logistic regression model was used to examine the correlations between various types of obesity and dyslipidemia. Given the potential for a non-linear relationship between BMI, WC, and dyslipidemia. The R software version 4.3.2 was utilized to develop a restricted cubic spline. The fundamental concept of a restricted cubic spline is to model the spline function, RCS(X), by strategically selecting the placement and number of nodes. This approach allows the continuous variable X to exhibit a smooth curve across the entire range of values. Following adjustment for confounding factors, three nodes of BMI and waist circumference (*P*_10_, *P*_50_, *P*_90_) were selected based on the Bayesian information criterion to investigate BMI and waist circumference among the different sexes. The dose–response relationship with the risk of dyslipidemia was explored. A two-sided test with a significance level of α = 0.05 was conducted.

## Results

### Study population

**Figure 1:**
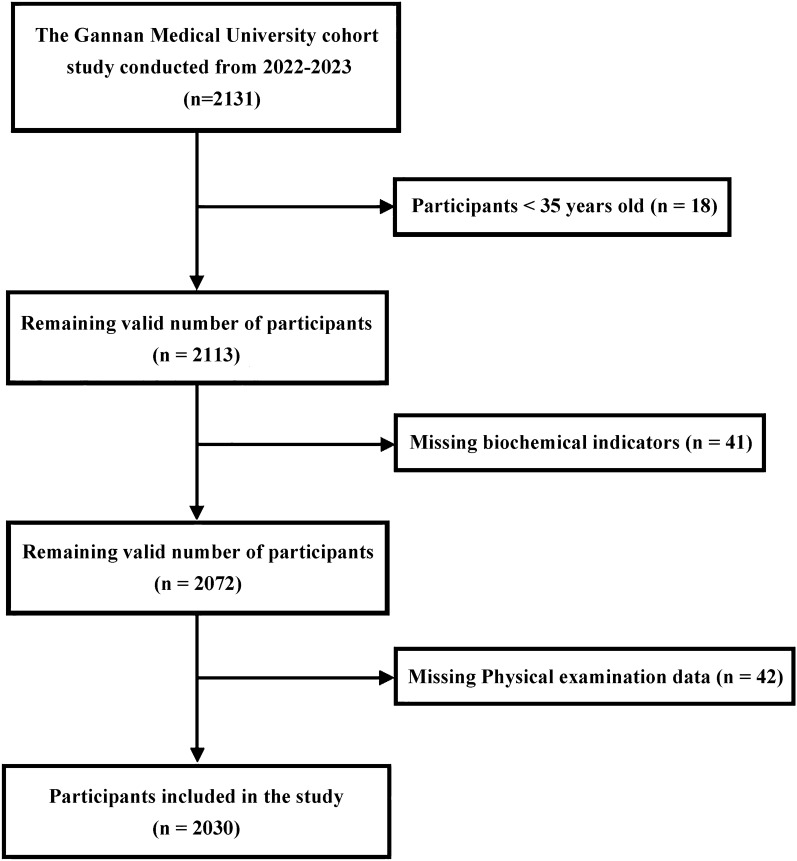
Flowchart of participants who were included in the study.

A total of 2131 individuals were surveyed in the Gannan Medical University Cohort from 2022 to 2023. After excluding individuals under 35 years old and those with incomplete data, the study included 2030 people aged 35 and above in Ganzhou, consisting of 637 males (31.38%) and 1,393 females (68.62%). The majority of participants were in the 50–64 age range, accounting for 1254 individuals (61.77%). Most had an educational level of junior high school or lower (70.74%). A vast majority were married, totaling 1,911 individuals (94.14%). The predominant occupation category was labeled as “other,” including 867 participants (42.71%) (Fig. 1, Table 1).

**Table 1:** Basic information of the survey population.

Variable	Subjects (*N* = 2030)	Proportion (%)/($\overline{x }\pm s$)
Sex		
Male	637	31.38
Female	1393	68.62
Age (years)		
35–49	325	16.01
50–64	1254	61.77
≥ 65	451	22.22
Education		
Primary school or lower	735	36.21
Middle school	701	34.53
High school	430	21.18
College or above	164	8.08
Marital status		
Married	1911	94.14
Other	119	5.86
Occupation		
Worker	314	15.47
Farmer	341	16.80
Cadre	48	2.36
Retired	460	22.66
Other	867	42.71
Family history		
Yes	1860	91.6
No	170	8.4
Diabetes history		
Yes	228	11.2
No	1802	88.8
Smoking status		
No smoke	1714	84.43
Smoking	189	9.31
Quit smoking	127	6.26
Drinking status		
No-drink	1492	73.50
Drinking	443	21.82
Quit drinking	95	4.68
Physical activity		
Low	309	15.22
Middle	923	45.47
High	798	39.31
BMI (kg/m^2^)	2030	23.74 $\pm$ 3.16
WC (cm)	2030	83.39 $\pm$ 9.10
TC (mmol/L)	2030	5.02 $\pm$ 0.92
TG (mmol/L)	2030	1.90 $\pm$ 1.36
LDL-L (mmol/L)	2030	3.04 $\pm$ 0.76
HDL-L (mmol/L)	2030	1.28 $\pm$ 0.28
FPG (mmol/L)	2030	5.36 $\pm$ 1.48
HbA1c	2030	5.49 $\pm$ 0.86

### Distribution of different obesity types

Central obesity was the predominant form of obesity among the participants, accounting for 28.87% (n = 586) of the population. The results of the χ^2^ test showed that sex, age, occupation, and educational level had a statistically significant impact on the distribution of obesity types (*P* < 0.05). However, marital status, smoking habits, and alcohol consumption did not significantly affect the distribution of obesity types (*P* > 0.05) (Table 2).

**Table 2:** General situation and distribution of different obesity types.

Variable	Non-obese *N*(*%*)	General obesity *N*(*%*)	Central obesity *N*(*%*)	Compound obesity *N*(*%*)	*χ*^*2*^	*P*
Sex					8.281	0.041
Male	385 (60.44)	3 (0.47)	181 (28.41)	68 (10.68)		
Female	882 (63.32)	9 (0.65)	405 (29.07)	97 (6.96)		
Age(years)					33.745	< 0.001
35–49	234 (72.00)	1 (0.31)	64 (19.69)	26 (8.00)		
50-64	787 (62.76)	11 (0.88)	356 (28.39)	100 (7.97)		
≥ 65	246 (54.55)	0 (0.0)	166 (36.81)	39 (8.65)		
Marital status					1.039	0.741
Married	1196 (62.59)	12 (0.63)	550 (28.78)	153 (8.01)		
Other	71 (59.66)	0 (0.00)	36 (30.25)	12 (10.08)		
Education						
Primary school or lower	425 (57.82)	9 (1.22)	230 (31.29)	71 (9.66)	26.410	0.002
Middle school	462 (65.91)	2 (0.29)	178 (25.39)	59 (8.42)		
High school	285 (66.28)	1 (0.23)	120 (27.91)	24 (5.58)		
College or above	95 (57.93)	0 (0.00)	58 (35.37)	11 (6.71)		
Occupation					33.153	< 0.001
Worker	197 (62.74)	5 (1.59)	81 (25.80)	31 (9.87)		
Farmer	204 (59.82)	4 (1.17)	95 (27.86)	38 (11.14)		
Cadre	24 (50.00)	0 (0.00)	19 (39.58)	5 (10.42)		
Retired	280 (60.87)	0 (0.00)	157 (34.13)	23 (5.00)		
Other	562 (64.82)	3 (0.35)	234 (26.99)	68 (7.84)		
Smoking status status					2.318	0.888
Non-smoke	1069 (62.37)	11 (0.64)	492 (28.70)	142 (8.28)		
Smoking	118 (62.43)	1 (0.53)	54 (28.57)	16 (8.47)		
Quit smoking	80 (62.99)	0 (0.00)	40 (31.50)	7 (5.51)		
Drinking status status					8.114	0.230
Non-drink	939 (62.94)	11 (0.74)	417 (27.95)	125 (8.38)		
Drinking	278 (62.75)	1 (0.23)	132 (29.80)	32 (7.22)		
Quit drinking	50 (52.63)	0 (0.00)	37 (38.95)	8 (8.42)		
T otal	1267 (62.41)	12 (0.59)	586 (28.87)	165 (8.13)		

### Prevalence and influencing factors of dyslipidemia

#### Prevalence of dyslipidemia and univariate analysis of each variable and dyslipidemia

In this study, 798 patients were diagnosed with dyslipidemia, resulting in a prevalence rate of 39.31%. After standardizing for the seventh census, the prevalence rate was adjusted to 36.51%. Statistical analysis using the *χ*^2^ test revealed significant differences in dyslipidemia prevalence based on sex, age, family history of dyslipidemia, history of diabetes, smoking status, and type of obesity (*P* < 0.05), whereas marital status, educational level, occupation, alcohol consumption, and physical activity did not show any statistically significant differences (*P* > 0.05). Among the different obesity types, the highest prevalence of dyslipidemia was observed in individuals with compound obesity (58.79%). (Table 3).

**Table 3:** Univariate analysis of variables and dyslipidaemia.

Variable	*N*(*%*)	Dyslipidemia		*χ*^*2*^	*P*
		*N*	Prevalence (*%*)		
Sex				14.282	< 0.001
Male	637 (31.38)	289	45.37		
Female	1393 (68.62)	509	36.54		
Age (years)				12.196	0.002
35–49	325 (16.01)	100	30.77		
50–64	1254 (61.77)	508	40.51		
≥ 65	451 (22.22)	190	42.13		
Marital status				0.002	0.966
Married	1911 (94.14)	751	39.30		
Other	119 (5.86)	47	39.50		
Education				1.913	0.591
Primary school or lower	735 (36.21)	284	38.64		
Middle school	701 (34.53)	267	38.09		
High school	430 (21.18)	180	41.86		
College or above	164 (8.08)	67	40.85		
Occupation				4.818	0.307
Worker	314 (15.47)	119	37.90		
Farmer	341 (16.80)	131	38.42		
Cadre	48 (2.36)	26	54.17		
Retired	460 (22.66)	181	39.35		
Other	867 (42.71)	341	39.33		
Family history				18.433	< 0.001
Yes	170 (8.4)	93	54.71		
No	1860 (91.6)	705	37.90		
Diabetes history				37.184	< 0.001
Yes	228 (11.23)	132	57.89		
No	1802 (88.77)	666	36.96		
Smoking status				11.556	0.003
No smoke	1714 (84.43)	649	37.86		
Smoking	189 (9.31)	95	50.26		
Quit smoking	127 (6.26)	54	42.52		
Drinking status				1.010	0.604
No-drink	1492 (73.50)	577	38.67		
Drinking	443 (21.82)	181	40.86		
Quit drinking	95 (4.68)	40	42.11		
Physical activity				2.135	0.344
Low	309 (15.22)	125	40.45		
Middle	923 (45.47)	375	40.63		
High	798 (39.31)	298	37.34		
Obesity types				81.221	< 0.001
Non-obese	1267 (62.41)	405	31.96		
General obesity	12 (0.59)	5	41.67		
Central obesity	586 (28.87)	291	49.66		
Compound obesity	165 (8.13)	97	58.79		

#### Logistic regression analysis of the risk of dyslipidemia in various obesity types

Univariate logistic regression analysis indicated that both central obesity and compound obesity were associated with an increased risk of dyslipidemia (central obesity: *OR* = 2.10, 95% CI 1.72–2.57; compound obesity: *OR* = 3.04, 95% CI 2.18–4.23). However, no significant relationship was observed between general obesity and dyslipidemia. After adjusting for confounding factors like adjusted for Sex, Age, Marital status, Education, Occupation, Family history, Diabetes history, Smoking status, Drinking status, Physical activity, logistic regression analysis still demonstrated that central obesity and compound obesity remained risk factors for dyslipidemia (central obesity: OR = 2.00, 95% CI 1.62–2.46; compound obesity: OR = 2.86, 95% CI 2.03–4.03). No significant association was found between general obesity and dyslipidemia (Table 4).

**Table 4:** Logistic regression analysis of various obesity types and dyslipidemia.

Obesity types	Unadjusted^a^		*Ρ*	Adjusted^b^		*Ρ*
	*OR*	95% CI		*OR*	95% CI	
Non-obese	1.00		< 0.001	1.00		< 0.001
General obesity	1.52	0.48–4.82	0.477	1.22	0.37–4.04	0.748
Central obesity	2.10	1.72–2.57	< 0.001	2.00	1.62–2.46	< 0.001
Compound obesity	3.04	2.18–4.23	< 0.001	2.86	2.03–4.03	< 0.001

a: unadjusted;

b: adjusted for Sex, Age, Marital statu, Education, Occupation, Family history, Diabetes history, Smoking status, Drinking status, Physical activity.

### Relationship between BMI and WC and risk of dyslipidemia by sex

#### Relationship between BMI and risk of dyslipidemia by sex

**Figure 2:**
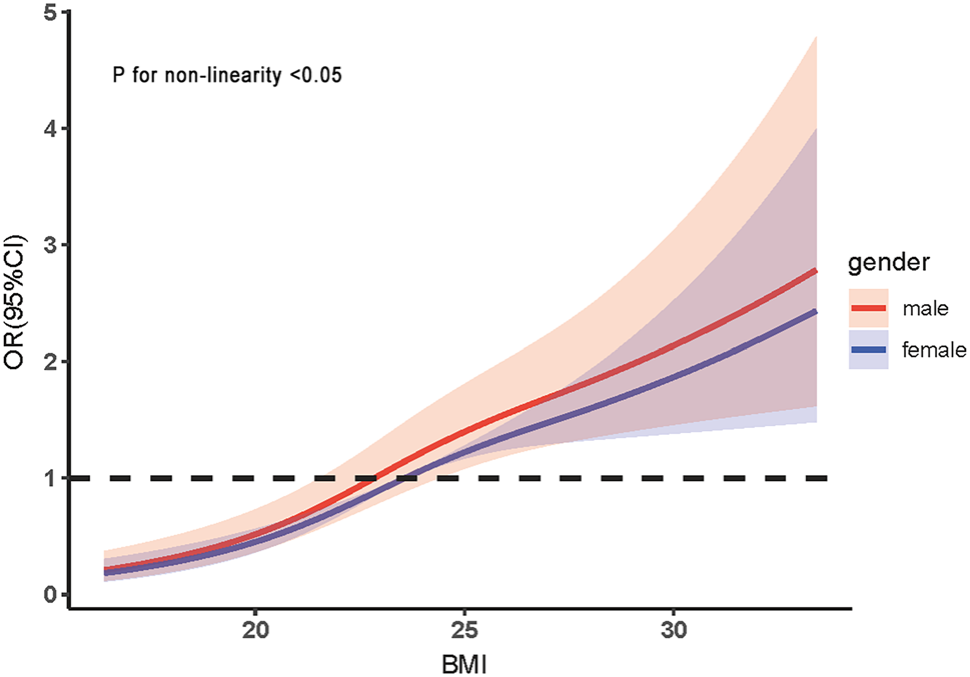
Dose–effect relationship between BMI and risk of dyslipidemia in different sex.

Logistic regression analysis indicated a positive association between increased BMI and a higher risk of dyslipidemia in both men (*OR* = 1.287, 95% CI 1.210–1.368) and women (*OR* = 1.102, 95% CI 1.062–1.145). Furthermore, age and lifestyle were identified as additional factors influencing this relationship, and after adjusting for confounding factors, a restricted cubic spline model was employed to investigate the dose–response correlation between BMI and dyslipidemia. The results indicated a predominantly linear connection between BMI and the likelihood of dyslipidemia in both male and female subjects, with a statistically significant nonlinear trend (*P* < 0.05). When BMI was 24.43 kg/m^2^ (*OR* = 1.30, 95% CI 1.01–1.68) in men and 23.66 kg/m^2^ (*OR* = 1.01, 95% CI 1.01–1.02) in women, there was a significant increase in the prevalence of dyslipidemia. Moreover, this risk was found to be higher in men than in women. (Fig. 2, Table 5).

**Table 5:** Relationship between BMI and risk of dyslipidemia by sex.

Variable	Male			Female		
	*OR*	95% CI	*Ρ*	*OR*	95% CI	*Ρ*
BMI	1.287	1.210–1.368	< 0.001	1.102	1.062–1.145	< 0.001
Age (years)			0.743			0.005
35–49	1.000					
50–64	1.212	0.710–2.068	0.481	1.709	1.200–2.434	0.003
≥ 65	1.105	0.573–2.129	0.765	2.009	1.280–3.153	0.002
Smoking status			0.008			1.000
No smoke	1.000			1.000		
Smoking	1.949	1.281–2.965	0.002	–	–	–
Quit smoking	1.352	0.834–2.192	0.221	–	–	–
Drinking status			0.963			0.606
No-drink	1.000			1.000		
Drinking	1.044	0.721–1.509	0.821	0.832	0.580–1.193	0.318
Quit drinking	1.069	0.570–2.004	0.835	0.963	0.480–1.934	0.916
Physical activity			0.159			0.856
Low	1.000			1.000		
Middle	0.938	0.570–1.546	0.803	0.966	0.685–1.360	0.318
High	0.672	0.403–1.121	0.128	0.915	0.646–1.297	0.916

Adjusted for Age, Marital status, Education, Occupation, Family history, Diabetes history, Smoking status, Drinking status, Physical activity.

#### Relationship between WC and risk of dyslipidemia by sex

**Figure 3:**
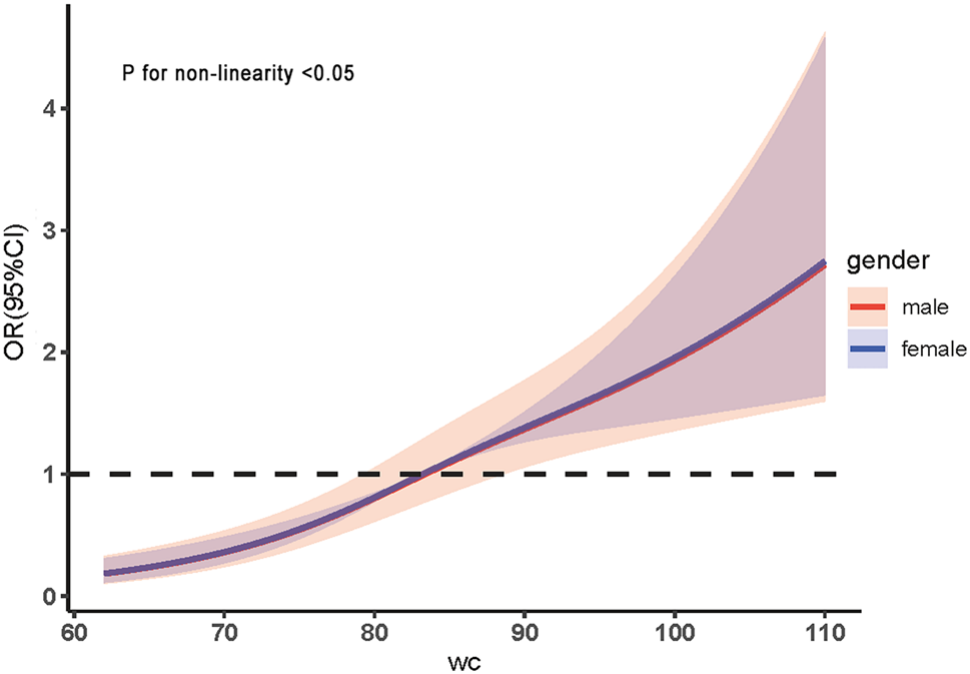
Dose–effect relationship between WC and risk of dyslipidemia in different sex.

Logistic regression analysis revealed a significant association between WC and risk of dyslipidemia in both men (*OR* = 1.087, 95% CI 1.065–1.110) and women (*OR* = 1.039, 95% CI 1.024–1.054). Moreover, this relationship was found to be influenced by factors such as age and lifestyle. Following adjustments for confounding factors, a restricted cubic spline model was employed to examine the dose–response association between WC and dyslipidemia. The findings indicated a predominantly linear correlation between waist circumference and the likelihood of dyslipidemia in both male and female participants, with a significant nonlinear test result (*P* < 0.05). When the WC was 88.77 cm in men (*OR* = 1.30, 95% CI 1.01–1.69) and 83.47 cm in women (*OR* = 1.02, 95% CI 1.01–1.02), there was a significant increase in the prevalence of dyslipidemia. Moreover, there was no significant difference in risk between men and women (Fig. 3, Table 6).

**Table 6:** Relationship between WC and risk of dyslipidemia by sex.

Variable	Male			Female		
	*OR*	95% CI	*Ρ*	*OR*	95% CI	*Ρ*
WC	1.087	1.065–1.110	< 0.001	1.039	1.024–1.054	< 0.001
Age(years)			0.702			0.028
35–49	1.000					
50–64	1.105	0.651–1.878	0.711	1.588	1.113–2.265	0.011
≥ 65	0.921	0.480–1.764	0.803	1.748	1.109–2.755	0.016
Smoking status			0.051			1.000
No smoke	1.000			1.000		
Smoking	1.671	1.105–2.526	0.015	–	–	–
Quit smoking	1.267	0.782–2.052	0.336	–	–	–
Drinking status			0.979			0.633
No-drink	1.000			1.000		
Drinking	0.964	0.668–1.392	0.845	0.846	0.590–1.213	0.363
Quit drinking	1.002	0.534–1.879	0.995	0.883	0.439–1.777	0.883
Physical activity			0.186			0.935
Low	1.000			1.000		
Middle	0.951	0.581–1.558	0.842	0.990	0.702–1.394	0.952
High	0.689	0.414–1.145	0.150	0.950	0.671–1.346	0.774

Adjusted for Age, Marital status, Education, Occupation, Family history, Diabetes history, Smoking status, Drinking status, Physical activity.

## Discussion

This cross-sectional study utilized BMI and WC as assessment criteria to categorize individuals aged 35 and older in Ganzhou into several body figure categories and explored the association between different types of obesity and the likelihood of developing dyslipidemia. Furthermore, a restricted cubic spline model was constructed to examine the dose–response correlation between BMI, WC, and the risk of dyslipidemia across various sexes. The findings revealed that central obesity (*OR* = 1.99, 95% CI 1.62–2.44) and compound obesity (*OR* = 2.84, 95% CI 2.02–3.97) were significant risk factors for dyslipidemia among residents in Ganzhou. Moreover, the restricted cubic spline model indicated that when BMI approached the overweight threshold and WC fell within the early stages of central obesity, there was a significant increase in the risk of dyslipidemia.

The findings revealed that the prevalence of dyslipidemia in Ganzhou was 39.31%, with an age-standardized prevalence of 36.51%. This prevalence was higher than the national average of 33.8% recorded from 2014 to 2019^10^ and the 33.8% observed among adults in Shaanxi Province in 2018^20^. However, it was lower than the 42.05% prevalence found among adult residents in Inner Mongolia^21^. Additionally, the prevalence rate among Tajik adults in Xinjiang was 37.0%^22^ and among adult residents in Qingdao in 2020 it was 40.53%^23^. The economic development level of Ganzhou City may intertwine with its unique Hakka culture, reflected in distinct cultural customs, eating habits, and lifestyles compared to other regions in the country. The prevalence of dyslipidemia in Ganzhou, possibly linked to the consumption of bacon and high-salt diets, highlights a significant health concern. To address this issue, the local government could consider implementing the ‘three reductions and three healthy conditions’ policies advocated by the National Health Commission, tailoring interventions to align with local Hakka culture and dietary practices to promote a healthier lifestyle for residents. Among residents aged over 35 years in Ganzhou, the prevalence rates of dyslipidemia were 41.67%, 49.66%, and 58.79% for general, central, and compound obesity, respectively. Notably, the prevalence of dyslipidemia among individuals with compound obesity was 58.79%. This aligns with findings from a survey by Zhang Milei et al. in Nanning, Guangxi^24^, underscoring the importance of addressing different types of obesity and implementing targeted intervention strategies, particularly for individuals with compound obesity.

This study suggests that individuals with compound obesity have a higher risk of dyslipidemia than non-obese individuals. Research by Huang et al.^25^ in Yangzhong City found that compound obesity posed the highest risk of dyslipidemia among residents aged 40–69. Similarly, a study of college students in Wuhu^26^ revealed that both compound and central obesity increased the risk of cardiovascular disease and metabolic abnormalities in adolescents, with compound obesity showing the highest risk. The aggregation of risk factors in individuals with compound obesity may contribute to this heightened risk^27^. Moreover, the interaction between general and central obesity could exacerbate the impact of compound obesity on dyslipidemia. Therefore, it is important to consider both BMI and WC during disease screening and prevention. Notably, this study did not find a significant association between general obesity and the risk of dyslipidemia, which could be attributed to the limited number of individuals with general obesity in the study population.

This study revealed that 37.59% of the residents in Ganzhou experienced various obesity issues, with a predominant occurrence of excessive WC. The findings from the restricted cubic spline model indicated a significant increase in the prevalence of dyslipidemia among individuals with pre-central obesity. Notably, there was no apparent disparity in dyslipidemia risk between sexes. Research has highlighted that individuals with central obesity tend to produce high levels of free fatty acids due to visceral fat accumulation, thereby promoting TG synthesis^28,29^. Additionally, visceral fat accumulation can alter lipase activity, enhance cholesterol synthesis, and contribute to the development of dyslipidemia. Moreover, the risk of dyslipidemia was notably elevated in men with a BMI of 24.43 kg/m^2^ and in women with a BMI of 23.66 kg/m^2^ compared to those with a normal BMI, with men exhibiting a higher risk than women. Numerous studies have demonstrated that being overweight or obese significantly increases the risk of dyslipidemia^30,31^. The observed sex differences could be attributed to the effect of estrogen on fat metabolism regulation and its role in inhibiting waist fat accumulation, potentially aiding women in reducing the risk of dyslipidemia associated with BMI^32,33^. The association between female BMI, WC, and the risk of dyslipidemia has been found to vary with age, and previous research has indicated that the impact of female BMI on TG and HDL-C levels changes as individuals grow older. Conversely, the relationship between male BMI, TG, and HDL-C levels remained consistent across different age groups, which aligns with the findings of this study^34^. Additionally, the link between male BMI, WC, and dyslipidemia risk is influenced by smoking habits, as tobacco smoke contains harmful substances such as nicotine, which can elevate free fatty acids in the bloodstream. This, in turn, promotes the synthesis of TG in the liver while reducing the activity of lipoprotein lipase, ultimately leading to higher TG levels and decreased HDL-C production^35^.

Multivariable regression analysis revealed no significant association between overall adiposity and dyslipidemia, contradicting the findings from the restricted cubic spline analysis. This study posits that the lower prevalence of general obesity among residents in southern Jiangxi may explain the lack of correlation between general obesity and dyslipidemia, suggesting that the type of obesity is influenced by both BMI and WC. The restricted cubic spline analysis specifically examines the relationship between BMI and dyslipidemia, revealing a non-linear association. Additionally, the logistic regression results for individual BMI and WC variables were in agreement with the findings from the restricted cubic spline analysis.

This study had several limitations. First, as a cross-sectional study, it could not establish a causal relationship between obesity type and dyslipidemia. Second, despite considering confounding variables such as general information, behavioral habits, and disease history, other influencing factors might still be unaccounted for. Thirdly, the sample size of individuals with general obesity in the study population is small, which might explain the lack of a significant association with dyslipidemia. However, this study addresses this limitation by utilizing a restricted cubic spline model to explore the dose–response relationship between BMI, WC, and the risk of dyslipidemia. This approach compensates for potential information loss due to the artificial classification of BMI and WC levels. Additionally, logistic regression was employed to adjust outcome-related parameters, enhancing the accuracy and objectivity of the final results. These findings provide valuable insights for the residents of southern Jiangxi seeking effective ways to prevent and manage dyslipidemia.

The prevalence of dyslipidemia and obesity among adult residents aged over 35 years in Ganzhou is a significant concern. Moreover, individuals with obesity and dyslipidemia face higher health risks. It is crucial to emphasize the importance of monitoring BMI and WC during disease screening and prevention.

## Data Availability

The data used in the current study can be obtained with reasonable justification from the corresponding authors.
